# *FBXW7* and Its Downstream *NOTCH* Pathway Could be Potential Indicators of Organ-Free Metastasis in Colorectal Cancer

**DOI:** 10.3389/fonc.2021.783564

**Published:** 2022-05-27

**Authors:** Dongzheng Li, Shiye Jiang, Xin Zhou, Chengshuai Si, Peng Shao, Qian Jiang, Liuqing Zhu, Lu Shen, Qi Meng, Jiani C. Yin, Yang Shao, Yueming Sun, Liu Yang

**Affiliations:** ^1^ Division of Colorectal Surgery, Department of General Surgery, The Affiliated Cancer Hospital of Nanjing Medical University & Jiangsu Cancer Hospital & Jiangsu Institute of Cancer Research, Nanjing, China; ^2^ Nanjing Geneseeq Technology Inc., Nanjing, China; ^3^ School of Public Health, Nanjing Medical University, Nanjing, China; ^4^ Division of Colorectal Surgery, Department of General Surgery, The First Affiliated Hospital of Nanjing Medical University, Nanjing, China & The First School of Clinical Medicine, Nanjing Medical University, Nanjing, China

**Keywords:** colorectal cancer, organ metastasis, *FBXW7*, *NOTCH*, NGS

## Abstract

Colorectal cancer (CRC) is one of the leading causes of cancer-related deaths globally. Metastasis is associated with a poor prognosis, yet the underlying molecular mechanism(s) remained largely unknown. In this study, a total of 85 CRC patients were included and the primary tumor lesions were evaluated by next-generation sequencing using a targeted panel for genetic aberrations. Patients were sub-divided according to their metastasis pattern into the non-organ metastases (Non-OM) and organ metastases (OM) groups. By comparing the genetic differences between the two groups, we found that mutations in *FBXW7* and alterations in its downstream *NOTCH* signaling pathway were more common in the Non-OM group. Moreover, correlation analysis suggested that *FBXW7* mutations were independent of other somatic alterations. The negative associations of alterations in *FBXW7* and its downstream *NOTCH* signaling pathway with CRC organ metastasis were validated in a cohort of 230 patients in the TCGA CRC dataset. Thus, we speculated that the genomic alterations of *FBXW7*/*NOTCH* axis might be an independent negative indicator of CRC organ metastases.

## Introduction

Colorectal cancer (CRC) is the second most common cause for cancer-related deaths and the fourth most commonly occurring malignant tumor worldwide (1). The main reason for poor treatment outcomes in CRC patients lies in the distant spread of cancer cells and organ metastasis could be the major cause for death (2–5). Thus, early interventions preventing the initial spread and occurrence of multiple metastases are crucial to improve CRC patient survival benefits (6, 7). However, the molecular mechanisms concerning the initiation, growth, spread, and distant metastasis of colorectal cancer are particularly complicated and partially unknown (8).

Universally, most of the CRC originated with the loss of function of the reputed gatekeeper gene *APC* and resulting in the formation of adenomas (9, 10). As the adenomas are growing, *KRAS* or *BRAF* malfunction begins to emerge and cell reproductive capacity would be abnormally enhanced (11). Eventually, a considerable amount of genetic alterations would accumulate, namely, the mutations of *PIK3CA*, *SMAD4* or *TP53*, and would enable the adenomas to transform into malignant tumors (11, 12). Moreover, several key pathways underlie the initiation and development of CRC, namely, the RTK*-*RAS, PI3K, and WNT pathways (13–15).

Aside from these relatively well-known primary developmental processes of CRC, there is limited evidence demonstrating some genetic features that could affect the distant metastasis. The CRC cells would eventually acquire the ability to migrate and colonize a new site, as they continuously accumulate additional genetic alterations (16, 17). Some studies have elucidated the associations between specific gene mutations and metastasis (18). For instance, it has been shown that peritoneal, distant lymphatic, and central nervous system (CNS) metastasis favor CRC patients harboring the *BRAF* V600E mutations (19). Abnormal activation of the cytokine receptor *c-KIT*, which is usually suppressed in the CRC cells, would result in an increase in the metastatic potential of the cancer cells (20, 21). In addition, chromosome 18q deletion would result in the simultaneous hepatic metastasis in CRC (22). These pieces of literature implicated a close relation of genetic alterations with CRC metastasis and could be the theoretical basis for our research.

To get a better understanding of the intrinsic molecular features relating to the organ metastasis in CRC patients, we profiled the genomic alterations of 85 patients with or without organ metastasis by using targeted next-generation sequencing (NGS) of 425 cancer-relevant genes. By comparing the molecular features among different metastatic groups, we discovered several characteristics that may serve as indicators of CRC organ metastasis. In the meantime, a parallel analysis of a TCGA CRC cohort (n = 230) was performed to validate the results we obtained (23).

## Methods

### Patients and Samples

From January 2020 to June 2021, a total of 85 colorectal cancer patients with or without organ metastasis who underwent surgical resections in the Jiangsu Cancer Hospital were included in this study. Surgical samples of the primary lesion were collected for genomic sequencing tests. Matched peripheral blood samples were also profiled as germline controls. The clinicopathologic features of each patient were retrospectively reviewed. All patients provided informed consents for taking part in this study.

### DNA Isolation, Library Construction and Sequencing Process

The tissue and blood samples were tested in a CLIA- and CAP-accredited central laboratory (Nanjing Geneseeq Technology Inc., Nanjing, China). In brief, genomic DNA (gDNA) was extracted from formalin-fixed paraffin-embedded tissue samples by using QIAamp DNA FFPE Tissue Kit (Qiagen) according to the manufacturer’s protocol. Peripheral blood was centrifuged at 1,900*g* for 10 min and gDNA from the white blood cells was also extracted as negative germline control. DNA quality was examined using the NanoDrop 2000 spectrophotometer (Thermo Fisher Scientific Waltham, MA, USA), and DNA quantification was performed on the Qubit 3.0 Fluorometer (Thermo Fisher Scientific, Waltham, MA, USA) with the dsDNA HS Assay Kit (Life Technologies, Carlsbad, CA, USA). The purified DNA was fragmented, and DNA libraries were constructed with the KAPA Hyper Prep kit (KAPA Biosystems) according to the manufacturer’s instruction. A panel targeting 425 cancer related genes (GeneseeqPrime™) using customized xGen lockdown probes (Integrated DNA Technologies) was used for hybridization capture. The hybridization reaction was performed with Dynabeads M-279 (Life Technologies) and xGen lockdown hybridization and wash kit (Integrated DNA Technologies). The target-enriched libraries were sequenced on the Illumina Hiseq4000 NGS platform (Illumina, San Diego, CA, USA) using the 150 bp paired-end reading according to the manufacturer’s protocol. The average coverage depth for the tumor tissue and the white blood cells were 1,000× and 60× respectively.

### Sequence Data Analysis

The sequence data analysis was carried out as previously described (24).In brief, the adapter reads, low quality reads and N bases were trimmed from the raw FASTQ files using Trimmomatic (25). Paired-end reads were aligned to the reference human genome (hg19). PCR deduplication was performed with Picard (http://broadinstitute.github.io/picard/). The base quality score recalibration and indel realignment were performed using the Genome Analysis Tool Kit (25). Somatic single nucleotide variants (SNVs) and small insertion/deletions (indels) were called by VarScan2 (26). Sequencing results from the tissue samples were compared with the matched white blood cell to identify the somatic mutations. SNVs and INDELs were further filtered using the following criteria: i) minimum ≥5 variant supporting reads and variant allele fraction (VAF) of ≥1%, ii) filtered out if present in >1% population frequency in the 1000 Genomes Project or ExAC database, iii) filtered out through an internal database of recurrent sequencing errors (≥3 variant reads and ≤20% VAF in at least 30 out of 2,000 normal control samples) on the same sequencing platform. Copy number variation (CNV) was screened using CNV kit. A fold change of more than 1.50 was considered to be a copy number gain and less than 0.65 was defined as copy number loss. Tumor mutational burden (TMB) was defined as the total number of base substitutions and indels in the coding region of targeted genes, namely, synonymous variants to reduce sampling noise and excluding known driver mutations as they are over-represented in the Panel, as previously described (27, 28).

### Microsatellite Instability (MSI) Analysis

MSI analysis was performed as previously described (29). The stability of a total of 52 microsatellite sites covered by the 425-cancer gene panel with a minimum of 15 bp repeats, including the classic MSI sites BAT-25, BAT-26, NR-21, NR-24, and MONO-27, was estimated and compiled into an overall MSI score. A site is considered qualified if it is covered by at least 101× depth of coverage. A sample is identified as MSI if more than 40% of the qualified sites with at least 100× coverage displayed instability.

### Statistical Analysis

Comparisons of proportion between groups were carried out using the Fisher’s exact test. Correlation analyses among gene alterations in different group were calculated by Spearman’s rank test. A two-sided P-value of <0.05 was considered statistically significant. All statistical analyses were performed using R (v.3.4.1).

## Results

### Patient Characteristics

Baseline clinicopathological features of the 85 CRC patients are summarized in **Table 1**. The median age of the study cohort was 55 years (range 29-78) and 50% were males. All patients were ECOG PS 0-1. Of these, 34 (40%) of patients had their primary lesions localized to the left colon, 23 (27.1%) right colon, and 12 (22.2%) rectum. The majority (77/85, 90.4%) of the cohort had adenocarcinoma, followed by the mixed histological type of six (7.1%) patients. Based on the status of metastasis, patients were subdivided into non-organ metastasis (Non-OM, n = 59) and organ-metastasis (OM, n = 26) groups. Notably, two (2.4%) cases were classified as signet-ring cell carcinoma. Both were stage IV CRC and belonged to the OM group, with metastasis to the very uncommon organ peritoneum plus bladder and peritoneum plus bone, respectively. Within the OM group, 20 (76.9%) were with liver metastasis, followed by 17 (65.4%) peritoneum metastasis, five (19.2%) lung metastasis and three other organ metastasis. Moreover, there are ten (38.5%), 13 (50%), and three (11.5%) cases possessing one, two, and three organ metastatic sites, respectively.

**Table 1:** Clinical characteristics of our cohort.

Characteristics	Non-OM (N = 59)	OM (N = 26)
**Age, years (median)**		
>55	28 (47.5%)	13 (50.0%)
≤55	31 (52.5%)	13 (50.0%)
**Sex**		
Female	33 (55.9%)	9 (34.6%)
Male	26 (44.1%)	17 (65.4%)
**Location**		
Left colon	24 (40.7%)	10 (38.5%)
Right colon	13 (22.0%)	10 (38.5%)
Rectum	22 (37.3%)	5 (19.2%)
Unknown	0 (0%)	1 (3.8%)
**Histological type**		
Adenocarcinoma	57 (96.6%)	20 (76.9%)
Mix	2 (3.4%)	4 (15.4%)
Signet-ring cell carcinoma	0 (0%)	2 (7.7%)
**Organ of metastasis**		
Liver	0 (0%)	20 (76.9%)
Peritoneum	0 (0%)	17 (65.4%)
Lung	0 (0%)	5 (19.2%)
Others	0 (0%)	3 (11.5%)
**Number of metastasis organ**		
0	59 (100%)	0
1	0	10 (38.5%)
2	0	13 (50.0%)
3	0	3 (11.5%)
**T**		
T1–T3	56 (94.9%)	13 (50%)
T4	3 (5.1%)	12 (46.2%)
TX	0	1 (3.8%)
**N**		
N0	26 (44.1%)	2 (7.7%)
N1–N2	33 (55.9%)	23 (88.5%)
NX	0	1 (3.8%)
**TMB**		
TMB-H	17 (28.8%)	5 (19.2%)
TMB-L	42 (71.2%)	21 (80.8%)
**MS**		
MSI-H	6 (10.2%)	1 (3.8%)
MSS	53 (89.8%)	25 (96.2%)

### Mutational Profile

**Figure 1:**
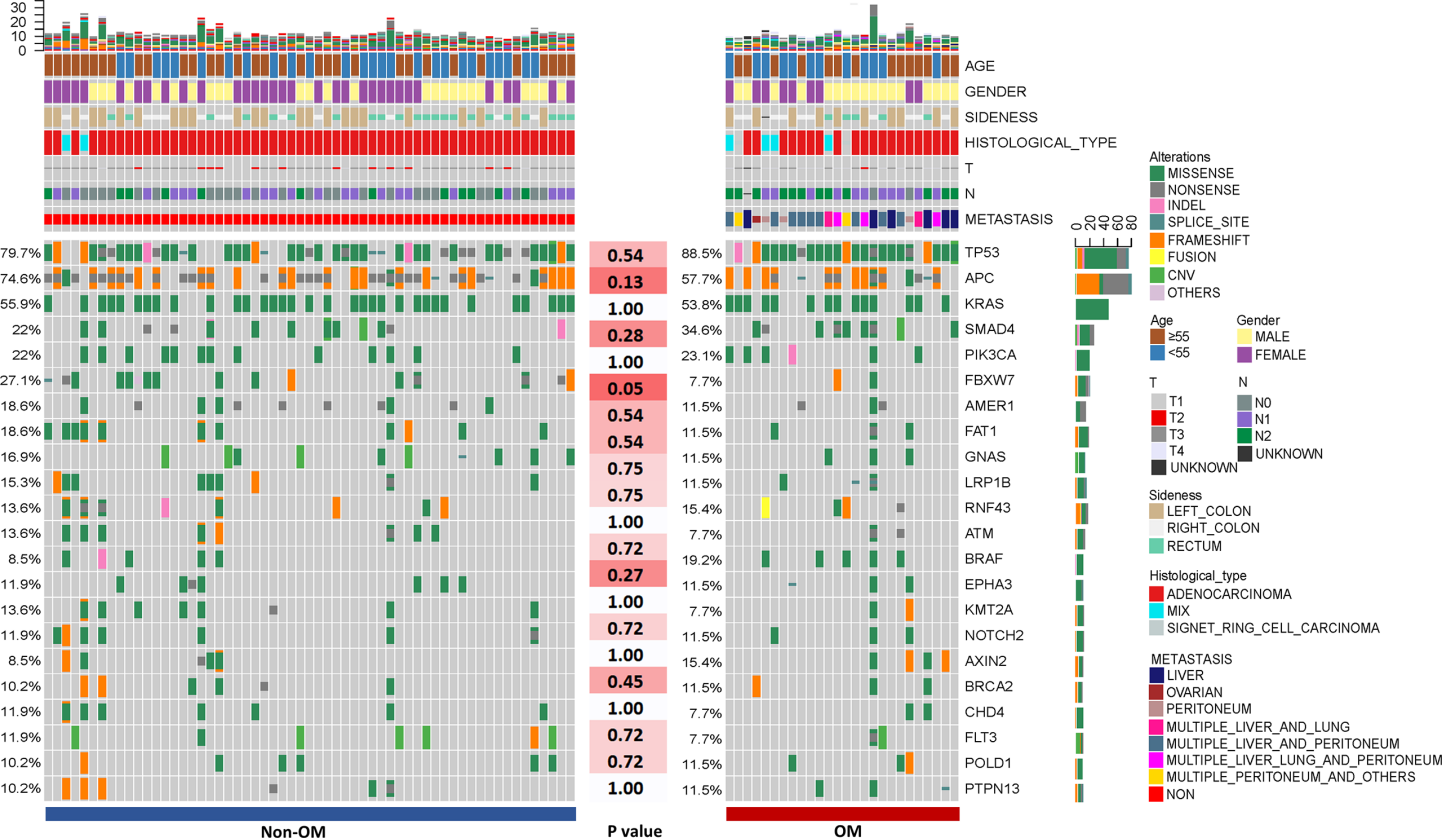
The clinical information and mutational spectrum of the colorectal patients in this study. Comparison of the mutational profile between the Non-OM and OM subgroups. The top 21 genes of the NGS cohort and P-values according to the Fisher’s exact were shown.

**Figure 2:**
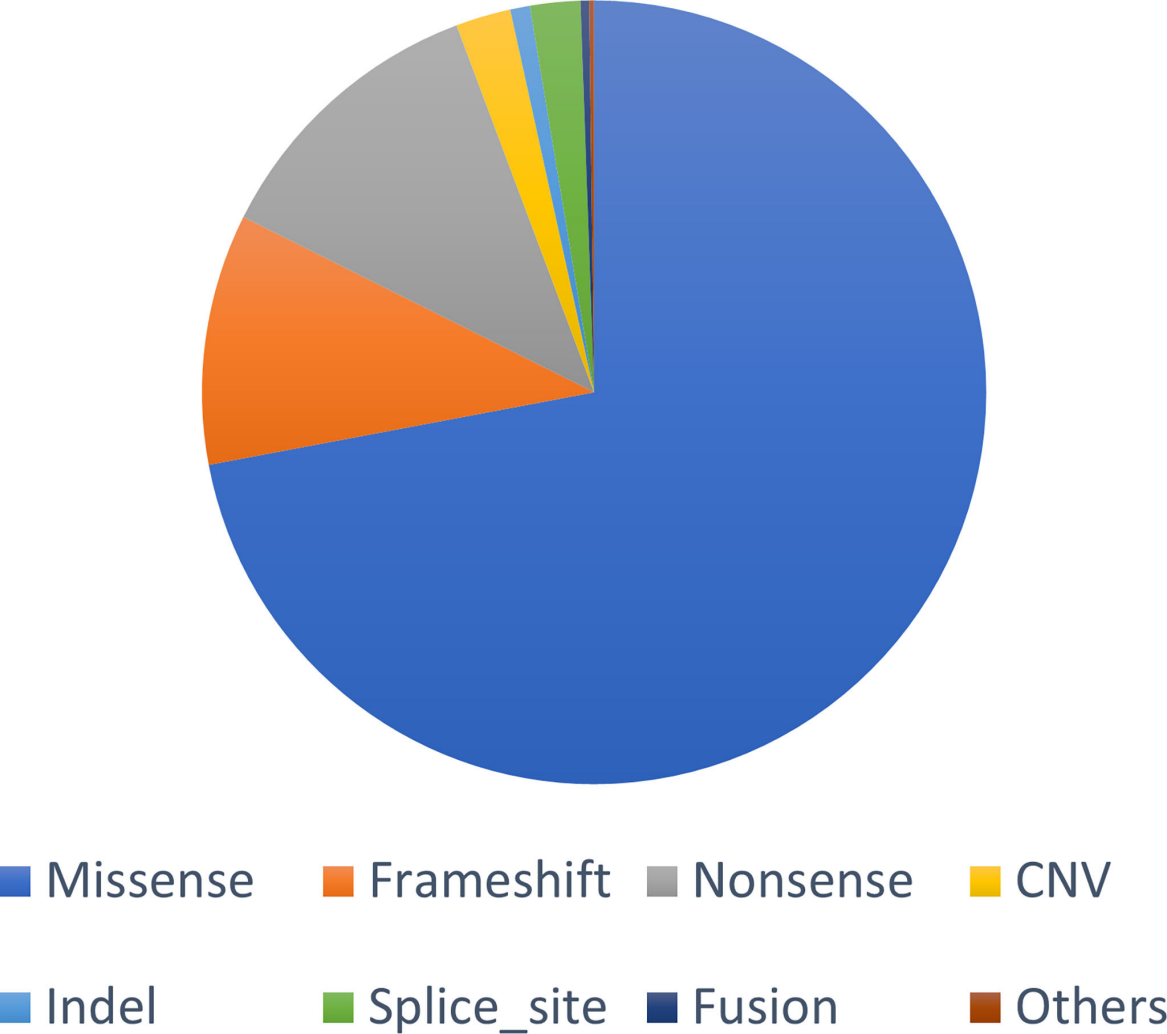
The somatic mutational type proportion of this cohort.

**Figure 3:**
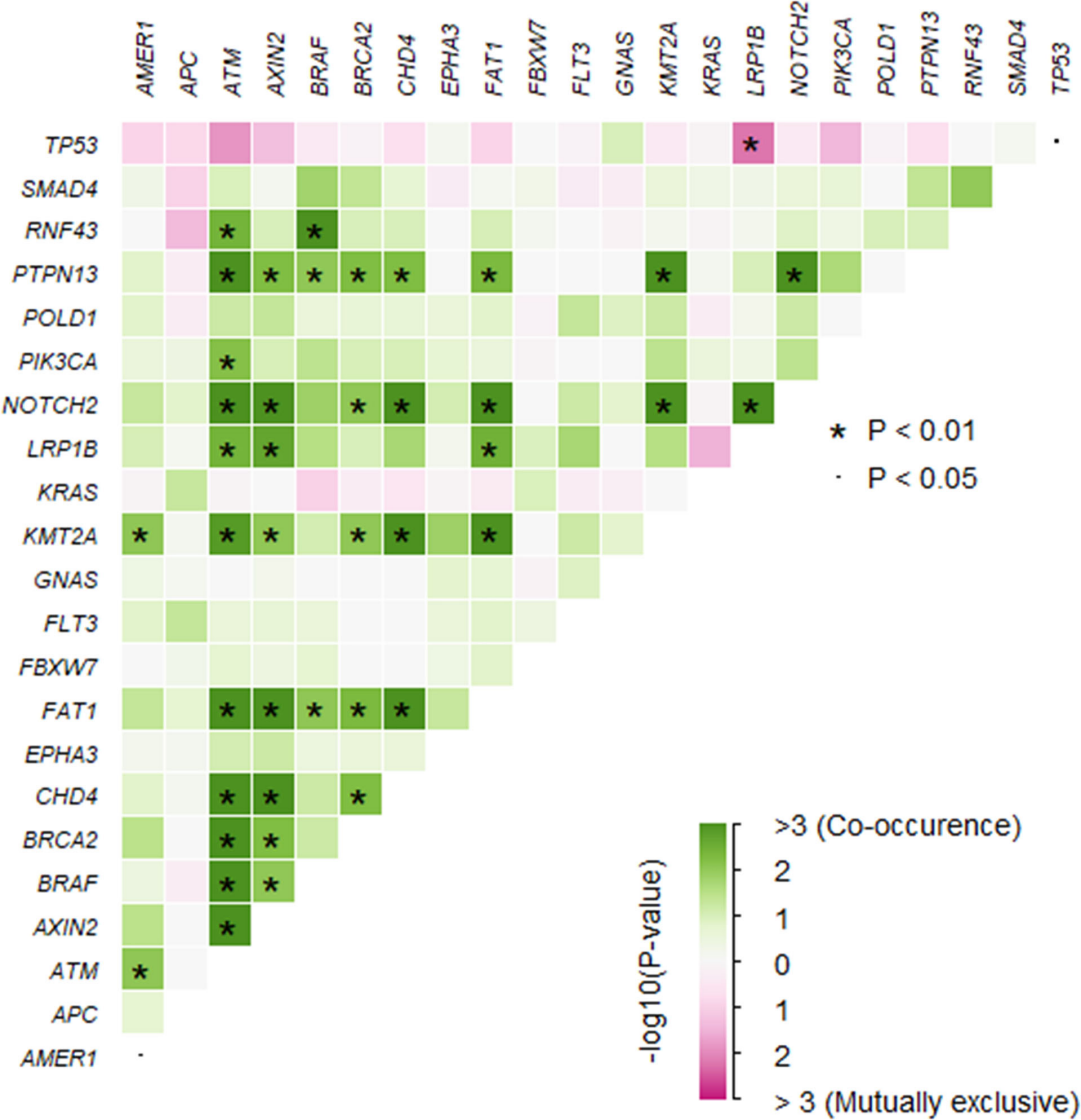
The somatic interactions of the study. Significant exclusive or co-occurrent variants were analyzed with Fisher’s exact test.

A total of 1,981 somatic mutations in 334 genes were detected. The top frequently altered genes in this cohort included *TP53* (82.4%), *APC* (69.4%), *KRAS* (55.3%), *SMAD4* (25.9%), *PIK3CA* (22.4%), and *FBXW7* (21.2%) (**Figure 1**). The majority of variant types were missense mutations (1,427/1,981, 72.0%), which was followed by nonsense mutations (236/1,981, 11.9%) and frame-shift mutations (205/1,981, 10.3%; **Figure 2**). The two most common mutated genes in CRC were analyzed. A total of 80 *TP53* alterations in 70 patients were detected (**Supplementary Figure S1A**). The p.R175H alteration was most common and 90% (72/80) of the alterations in *TP53* were oncogenic or likely oncogenic according to OncoKB annotation. Mutations in *LRP1B* were found in 12 (14.1%) patients, and were mutually exclusive with *TP53* alterations (P <0.01; **Figure 3**). Meanwhile, we detected 105 *APC* alterations belonging to 59 patients (**Supplementary Figure S1B**) and 88.6% (93/105) of the mutations were oncogenic and likely oncogenic.

Next, we examined the frequencies of oncogenic driver mutations in our cohort. Oncogenic mutations in *KRAS*, *NRAS*, and *BRAF* were detected in 55.3% (47/85), 7.1% (6/85), and 11.8% (10/85) of the cohort. *KRAS* G12 (82%, 41/50) was the most commonly mutated codon, and included G12D (30%, 15/50), G12V (16%, 8/50), G12R (8%, 4/85), G12C (6%, 3/50), G12A (4%, 2/50), and G12S (4%, 2/50) variants. The second most frequently altered codon was G13, consisting of G13D (14%, 7/50) and G13R (2%, 1/50). Furthermore, we also detected another eight (16%) types of *KRAS* variants located in the 2nd, 3rd, and 4th exons (**Supplementary Figure S1C**).

We also detected relatively high frequencies of alterations in *EGFR* (4.7%, 4/85), *ALK* (8.2%, 7/85), *MET* (2.4%, 2/85), *ROS1* (5.9%, 5/85), *ERBB2* (7.1%, 6/85), and *NTRK1/2/3* (10.1%, 10/85), which may potentially serve as targets for targeted therapies. None of these mutations were known oncogenic/targetable mutations, except for an irregular *SPTBN1*~*ALK* fusion gene that retained the complete kinase domain of *ALK*. The presence of *ALK* fusion suggested that CRC might benefit from ALK-targeted therapies.

### Associations Between Clinical Characteristics and Somatic Mutations

In addition, we performed mutational analysis comparing patients with various clinical characteristics. As compared to T1-3, T4 tumors had significantly higher mutational frequency in three genes, *BRAF* (5/15 vs. 5/69, p = 0.01), *IFNE* (2/15 vs. 0/69, p = 0.03), and *RNF43* (5/69 vs. 7/69, p = 0.03). By comparing the patients with and without lymph metastasis, we found that alteration frequencies of 79 genes were significantly different between two groups, of those six genes had mutational frequencies more than 10% in this cohort (**Supplementary Table 1**). Specifically, mutations of five genes favored non-lymph metastasis, namely *ATM* (8/28 vs. 2/56, p <0.01), *CHD4* (7/28 vs. 2/56, p <0.01), *AMER1* (9/28 vs. 5/56, p = 0.01), *KMT2A* (7/28 vs. 3/56, p = 0.01), and *NOTCH2* (7/28 vs. 3/56, p = 0.01). On the other hand, *TP53* mutations were enriched in the lymph metastasis group (19/28 vs. 51/56, p = 0.01). Moreover, a comparison between patients older or younger than median age was performed and showed that mutations in *KDR* (7/44 vs. 0/41, p = 0.01) were more common in the younger patients while mutations in *ARAF* (0/44 vs. 5/41, p = 0.02), *AXL* (0/44 vs. 5/41, p = 0.02) and *APC* (26/44 vs. 33/41, p = 0.04) were more common in the older patients. These results could potentially help to reveal the pathogenesis of CRC patients with diverse clinical features.

### Identification of Indicators in CRC Organ Metastasis

To identify potential genetic risk factors of organ metastasis, we analyzed the genetic features between the Non-OM and OM subgroups. To examine if the results were reliable, a TCGA CRC dataset (23) was used as a validation cohort.

To assess the influences of single gene aberrations on organ metastasis, we compared the genetic mutations (with mutational frequencies >10%) between the two groups. We observed a higher frequency of *FBXW7* alterations in Non-OM than that in the OM group (27.1% vs. 7.7%; p = 0.05; **Figure 1**), which was validated in the TCGA CRC dataset (Non-OM, 19.3%; OM, 0%; p <0.01; **Supplementary Figure S2A**). To further examine if *FBXW7* mutations act independently of other genes, we performed a mutual exclusivity or co-occurrence analysis. Notably, the *FBXW7* mutations occurred independently from mutations in other genes (**Figure 3**). Functional annotations of all the *FBXW7* variants with OncoKB revealed that the oncogenic or likely oncogenic *FBXW7* mutations were significantly enriched in the Non-OM group than that in the OM group (20.3% vs. 3.8%; p = 0.05; **Supplementary Figures S2B, C**; **Supplementary Table 2**). We also evaluated the functional variants in various oncogenic signaling pathways that might be related to CRC organ metastasis. Interestingly, the *NOTCH* pathway locating downstream of *FBXW7* was more frequently altered in the Non-OM than in the OM group in both our cohort (22% vs. 3.8%, p = 0.05) and the TCGA cohort (10.9% vs. 0, p = 0.05). Thus, we speculated that *FBXW7* and its downstream *NOTCH* pathway might play an independent and suppressive role in CRC organ metastasis.

In addition, we studied the distribution of all the altered genes in both our and the TCGA cohorts and found that there are 54 genes (all with mutational frequencies less than 10%) only mutated in the Non-OM groups of both cohorts but not in the OM groups. A pathway analysis implicated that these mutations were significantly enriched in several important cancer-related pathways, including the p38 MAPK cascade, response to cAMP, and leukocyte activation, etc. (**Supplementary Figure S3**). Thus, these pathways might play vital roles in the regulation/suppression of CRC organ metastases.

Apart from the feature of single genes and pathways, distinct mutational features were identified among different groups. We analyzed the mutational type proportion between the two groups. The results suggested that in both groups, the top common mutational type were missense, frame-shift and nonsense variants, with minor trends of differences between the Non-OM subgroup [68.4% (793/1,160), 13.7% (159/1,160), and 11.7% (136/1,160) respectively] and the OM subgroup [77.2% (634/821), 5.6% (46/821), and 12.2% (100/821) (**Supplementary Figure S4A**). Similar results were observed in the TCGA cohort (**Supplementary Figure S4B**). Moreover, the numbers of the somatic mutations of each patient between the groups were also investigated, though no significant difference was observed in either our cohort or the validation cohort (**Supplementary Figures S5A, B**).

Since there are evidences that microsatellite (MS) instable CRC and tumor mutation burden (TMB) high CRC may possess disparate biological characters (30, 31), we subsequently inspected the associations of TMB and MS status with respect to the organ metastasis status (**Table 1**). In our cohort, 22 patients had high TMB (TMB ≥10 mut/Mb), of which seven patients were MSI-H (**Supplementary Figure S6**). Given the moderate sample size, both the TMB-H (n = 22, 25.9%, p = 0.43) and MSI-H (n = 7, 8.2%, p = 0.43) patients showed no statistically significant associations with OM vs. Non-OM compared with the TMB-L and MSS patients, respectively (**Supplementary Figures S7A, B**). In *FBXW7*-mutated tumors, there was a trend towards higher TMB compared with those with wild-type *FBXW7* (p = 0.12; **Supplementary Figure S8**). In line with the previous result, within the TMB-L population, *FBXW7* mutations more commonly occurred in the Non-OM group than that of OM group (26.2% vs. 4.8%, p = 0.05). Interestingly, within the TMB-H population, it was *CTNNB1*, but not *FBXW7*, mutations that significantly differed between the non-OM and OM groups, with the frequency of 23.5% (4/17) in the Non-OM and 80% (4/5) in the OM (p = 0.04) groups. This finding suggested that TMB-H CRC and TMB-L CRC might be driven by distinct organ metastatic mechanisms. Due to the low number of the MSI-H cases, we could not perform the similar analysis.

## Discussion

In the past decades, efforts have been made to discover the molecular mechanisms underlying the organ metastasis in CRC. By comparing the genomic aberrations in different metastatic groups, previous studies have discovered a number of key genes and pathways that function to regulate the spread and colonization of CRC cells to the distant organs. In this work, we investigated 425 cancer-related genes in a cohort of 85 CRC patients and an independent validation cohort of 230 patients in the TCGA CRC dataset. By comparing the genetic features of Non-OM and OM group, we discovered that the mutations in *FBXW7*, as well as its downstream *NOTCH* signaling pathway genes, were significantly enriched in the CRC patients without organ metastasis, which was validated in the TCGA cohort. In accordance with our finding, *FBXW7* mutations have been reported to exhibit a tendency more commonly appeared in early-stage CRC patients (32, 33).

The frequency of *FBXW7* mutations in CRC is around 6 to 10% (32). *FBXW7* is a key participant in the ubiquitin-proteosome system and negatively regulates the downstream cancer-related genes (34). Mounting studies have demonstrated that *FBXW7* plays critical roles in tumor initiation, cell proliferation, cell differentiation and angiogenesis (35–37). In line with this, it has been reported that some missense mutations in *FBXW7* could enhance cell proliferation, migration and invasion in cervical cancer cells (38). Previous reports have suggested that *FBXW7* mutations could improve cancer-initiating cell activities through the *NOTCH* signaling pathways (39, 40). In our study, we found that not only *FBXW7* mutations but also alterations in its downstream *NOTCH* pathway were significantly enriched in the Non-OM group. This phenomenon was also validated in the TCGA CRC cohort. Therefore, the *FBXW7*/*NOTCH* regulating axis might play an important role in the organ metastasis of CRC. In summary, we speculate that *FBXW7* and its downstream *NOTCH* pathway could be the indicators for CRC organ metastasis.

It is worth pointing out that all patients in the Non-OM group presented with early stage CRC at the time of diagnosis. As a consequence, due to the retrospective nature of our study, we cannot fully differentiate whether the association of *FBXW7* mutations were OM-specific or could be related to tumor stage at disease presentation. In addition, while our findings likely support the positive prognostic value of *FBXW7* mutations in CRC patients, future investigations are needed to demonstrate the association between FBXW7 mutation and CRC prognosis.

A discrepancy of the result is that *FBXW7* is a cancer suppressor gene and there are proofs that mutation of *FBXW7* can increase the degree of malignancy of tumor cells (36, 38, 41). However, in our cohort, the TCGA cohort and in other pieces of literature the alterations in *FBXW7* more frequently occurred in early stage CRC patients (32, 33). We think that it might be because that *FBXW7* is a central regulator in cancer biology mediating a diverse array of oncogenic processes. As a consequence, alterations in *FBXW7* might have distinct consequences under different biological context. For example, it was reported that *FBXW7* mutation in a single allele would diminish the activity of *FBXW7* more severely than the bi-allelic or the homozygous mutation in the intestine of mice (33). Furthermore, *FBXW7* heterozygous mutations have been reported to exhibit a higher tumorigenic rate than homozygous mutations in mice (42). Thus, future studies are required to explain this seemingly paradoxical phenomenon.

It is well known that TMB-H and TMB-L CRC share highly distinct pathogenesis and would exhibit differential responses to various treatments (30, 43, 44). In this study, we also noticed that another gene *CTNNB1* but not *FBXW7* may be a more useful indicator for CRC metastasis in the TMB-H population. The *CTNNB1* variants were significantly enriched in the TMB-H OM group, compared with that of TMB-H Non-OM group. In addition, mutations in *CTNNB1* could irregularly activate the *WNT* pathway without the ligands and subsequently result in the abnormal growth of cells and development of cancer (45, 46). Thus, our data suggested that *CTNNB1* mutations could be an indicator of CRC metastasis in the TMB-H population.

In conclusion, by comparing the molecular differences among CRC patients with different metastasis statuses, we discovered that *FBXW7* and its downstream *NOTCH* pathway were more commonly mutated in the Non-OM group than in the OM group. Therefore, *FBXW7/NOTCH* regulating axis may play important roles in organ metastasis in CRC. Our study contributes to the understanding of the molecular mechanisms of CRC metastastic processes.

## Data Availability Statement

The original contributions presented in the study are publicly available. This data can be found here: https://ngdc.cncb.ac.cn/gsa-human/, HRA002412.

## Ethics Statement

The study has been approved by the Ethics committee of Jiangsu Cancer Hospital. All patients have provided the written consent forms.

## Author Contributions

Study concept and design: DL and YuS. Manuscript writing and revision: DL, LY, QM, and JY. NGS testing and analysis: LZ, LS, QM, JY, YaS. Clinical information collection: DL, SJ, XZ, LY. Pathological evaluation: CS, PS, QJ. All authors contributed to the article and approved the submitted version.

## Funding

This study was supported by the National Natural Science Foundation of China (81602145 and 82072704, to L.Y.), Jiangsu Primary Research & Development Plan (SBE2021740280), Jiangsu Provincial Natural Science Foundation (BK20171509 to L.Y.), Jiangsu Provincial Medical Youth Talent, The Project of Invigorating Health Care through Science, Technology Education (QNRC2016649 to L.Y.), the China Postdoctoral Science Foundation (2018M632265 to L.Y.), The “333 Talents” Program of Jiangsu Province (BRA2020390 to L.Y.) and The Talents Program of Jiangsu Cancer Hospital (2017-33 to L.Y.).

## Conflict of Interest

LZ, LS, QM, JY, and YS are the employees of Nanjing Geneseeq Technology Inc.

The remaining authors declare that the research was conducted in the absence of any commercial or financial relationships that could be construed as a potential conflict of interest.

## Publisher’s Note

All claims expressed in this article are solely those of the authors and do not necessarily represent those of their affiliated organizations, or those of the publisher, the editors and the reviewers. Any product that may be evaluated in this article, or claim that may be made by its manufacturer, is not guaranteed or endorsed by the publisher.

## Supplementary Material

Supplementary Figure 1 The distribution of the detected somatic variants in this study. **(A)**, *TP53*; **(B)**, *APC* and **(C)**, *KRAS*.

Supplementary Figure 2
**(A)** The mutational spectrum of the colorectal patients in TCGA cohort. Comparison of the mutational profile between the Non-OM and OM subgroup and the P values according to the Fisher’s exact test was shown. The distribution of the detected *FBXW7* variants in the non-OM **(B)** and OM **(C)** groups.

Supplementary Figure 3 The distribution of the mutated genes in both this study and TCGA cohort with or without organ metastasis **(A)**. The GO pathway analysis of the 54 genes that only distributed in the OM groups **(B)**.

Supplementary Figure 4 The mutational type proportion of Non-OM and OM groups in this study **(A)** and TCGA cohort **(B)**.

Supplementary Figure 5 The numbers of the somatic mutations of each patient in the Non-OM and OM groups of this study **(A)** and TCGA cohort **(B)**.

Supplementary Figure 6 The interaction of TMB and MS status in this study.

Supplementary Figure 7 The TMB **(A)** and MSI **(B)** distribution in the Non-OM and OM groups of this study.

Supplementary Figure 8 The comparison of TMB between *FBXW7* mutated and WT.

## References

[1] Bray F Ferlay J Soerjomataram I Siegel RL Torre LA Jemal A. Global Cancer Statistics 2018: GLOBOCAN Estimates of Incidence and Mortality Worldwide for 36 Cancers in 185 Countries. CA Cancer J Clin (2018) 68:394–424. doi: 10.3322/caac.21492 30207593

[2] Hanahan D Weinberg RA. Hallmarks of Cancer: The Next Generation. Cell (2011) 144:646–74. doi: 10.1016/j.cell.2011.02.013 21376230

[3] Valastyan S Weinberg RA. Tumor Metastasis: Molecular Insights and Evolving Paradigms. Cell (2011) 147:275–92. doi: 10.1016/j.cell.2011.09.024 PMC3261217 22000009

[4] Croner RS Fortsch T Bruckl WM Rodel F Rodel C Papadopoulos T. Molecular Signature for Lymphatic Metastasis in Colorectal Carcinomas. Ann Surg (2008) 247:803–10. doi: 10.1097/SLA.0b013e31816bcd49 18438117

[5] Pretzsch E Bosch F Neumann J Ganschow P Bazhin A Guba M. Mechanisms of Metastasis in Colorectal Cancer and Metastatic Organotropism: Hematogenous Versus Peritoneal Spread. J Oncol (2019) 2019:7407190. doi: 10.1155/2019/7407190 31641356 PMC6770301

[6] Duan L Yang W Wang X Zhou W Zhang Y Liu J. Advances in Prognostic Markers for Colorectal Cancer(). Expert Rev Mol Diagnostics (2019) 19:313–24. doi: 10.1080/14737159.2019.1592679 30907673

[7] Walther A Johnstone E Swanton C Midgley R Tomlinson I Kerr D. Genetic Prognostic and Predictive Markers in Colorectal Cancer. Nat Rev Cancer (2009) 9:489–99. doi: 10.1038/nrc2645 19536109

[8] Pappas-Gogos G Baltagiannis EG Kyrochristos ID Ziogas DE Goussia A Mitsis M. Predictive and Patient-Monitoring Biomarkers: Precision in the Management of Colorectal Cancer. Biomarkers Med (2020) 14:335–9. doi: 10.2217/bmm-2020-0025 32250157

[9] Kinzler KW Vogelstein B. Cancer-Susceptibility Genes. Gatekeepers and Caretakers. Nature (1997) 386:761, 763. doi: 10.1038/386761a0 9126728

[10] Aghabozorgi AS Bahreyni A Soleimani A Bahrami A Khazaei M Ferns GA. Role of Adenomatous Polyposis Coli (APC) Gene Mutations in the Pathogenesis of Colorectal Cancer; Current Status and Perspectives. Biochimie (2019) 157:64–71. doi: 10.1016/j.biochi.2018.11.003 30414835

[11] Li ZN Zhao L Yu LF Wei MJ. BRAF and KRAS Mutations in Metastatic Colorectal Cancer: Future Perspectives for Personalized Therapy. Gastroenterol Rep (2020) 8:192–205. doi: 10.1093/gastro/goaa022 PMC7333923 32665851

[12] Fearon ER Vogelstein B. A Genetic Model for Colorectal Tumorigenesis. Cell (1990) 61:759–67. doi: 10.1016/0092-8674(90)90186-i 2188735

[13] Steeg PS. Targeting Metastasis. Nat Rev Cancer (2016) 16:201–18. doi: 10.1038/nrc.2016.25 PMC7055530 27009393

[14] Bailey MH Tokheim C Porta-Pardo E Sengupta S Bertrand D Weerasinghe A. Comprehensive Characterization of Cancer Driver Genes and Mutations. Cell (2018) 173:371–385 e318. doi: 10.1016/j.cell.2018.02.060 29625053 PMC6029450

[15] Nie X Liu H Liu L Wang YD Chen WD. Emerging Roles of Wnt Ligands in Human Colorectal Cancer. Front Oncol (2020) 10:1341. doi: 10.3389/fonc.2020.01341 32923386 PMC7456893

[16] Vermaat JS Nijman IJ Koudijs MJ Gerritse FL Scherer SJ Mokry M. Primary Colorectal Cancers and Their Subsequent Hepatic Metastases Are Genetically Different: Implications for Selection of Patients for Targeted Treatment. Clin Cancer Res (2012) 18:688–99. doi: 10.1158/1078-0432.CCR-11-1965 22173549

[17] Massague J Obenauf AC. Metastatic Colonization by Circulating Tumour Cells. Nature (2016) 529:298–306. doi: 10.1038/nature17038 26791720 PMC5029466

[18] Tan IB Malik S Ramnarayanan K McPherson JR Ho DL Suzuki Y. High-Depth Sequencing of Over 750 Genes Supports Linear Progression of Primary Tumors and Metastases in Most Patients With Liver-Limited Metastatic Colorectal Cancer. Genome Biol (2015) 16:32. doi: 10.1186/s13059-015-0589-1 25808843 PMC4365969

[19] Morris V Overman MJ Jiang ZQ Garrett C Agarwal S Eng C. Progression-Free Survival Remains Poor Over Sequential Lines of Systemic Therapy in Patients With BRAF-Mutated Colorectal Cancer. Clin Colorectal Cancer (2014) 13:164–71. doi: 10.1016/j.clcc.2014.06.001 PMC4266576 25069797

[20] Ishaque N Abba ML Hauser C Patil N Paramasivam N Huebschmann D. Whole Genome Sequencing Puts Forward Hypotheses on Metastasis Evolution and Therapy in Colorectal Cancer. Nat Commun (2018) 9:4782. doi: 10.1038/s41467-018-07041-z 30429477 PMC6235880

[21] Gavert N Shvab A Sheffer M Ben-Shmuel A Haase G Bakos E. C-Kit Is Suppressed in Human Colon Cancer Tissue and Contributes to L1-Mediated Metastasis. Cancer Res (2013) 73:5754–63. doi: 10.1158/0008-5472.CAN-13-0576 24008320

[22] Tanaka T Watanabe T Kitayama J Kanazawa T Kazama Y Tanaka J. Chromosome 18q Deletion as a Novel Molecular Predictor for Colorectal Cancer With Simultaneous Hepatic Metastasis. Diagn Mol Pathol Am J Surg Pathol Part B (2009) 18:219–25. doi: 10.1097/PDM.0b013e3181910f17 19861895

[23] The Cancer Genome Atlas Network. Comprehensive Molecular Characterization of Human Colon and Rectal Cancer. Nature (2012) 487:330–7. doi: 10.1038/nature11252 PMC3401966 22810696

[24] Zhou Z Liu Z Ou Q Wu X Wang X Shao Y. Targeting FGFR in Non-Small Cell Lung Cancer: Implications From the Landscape of Clinically Actionable Aberrations of FGFR Kinases. Cancer Biol Med (2021) 18:490–501. doi: 10.20892/j.issn.2095-3941.2020.0120 PMC8185861 33710807

[25] McKenna A Hanna M Banks E Sivachenko A Cibulskis K Kernytsky A. The Genome Analysis Toolkit: A MapReduce Framework for Analyzing Next-Generation DNA Sequencing Data. Genome Res (2010) 20:1297–303. doi: 10.1101/gr.107524.110 PMC2928508 20644199

[26] Koboldt DC Zhang Q Larson DE Shen D McLellan MD Lin L. VarScan 2: Somatic Mutation and Copy Number Alteration Discovery in Cancer by Exome Sequencing. Genome Res (2012) 22:568–76. doi: 10.1101/gr.129684.111 PMC3290792 22300766

[27] Fang W Ma Y Yin JC Hong S Zhou H Wang A. Comprehensive Genomic Profiling Identifies Novel Genetic Predictors of Response to Anti-PD-(L)1 Therapies in Non-Small Cell Lung Cancer. Clin Cancer Res (2019) 25:5015–26. doi: 10.1158/1078-0432.CCR-19-0585 31085721

[28] Chalmers ZR Connelly CF Fabrizio D Gay L Ali SM Ennis R. Analysis of 100,000 Human Cancer Genomes Reveals the Landscape of Tumor Mutational Burden. Genome Med (2017) 9:34. doi: 10.1186/s13073-017-0424-2 28420421 PMC5395719

[29] Ying J Yang L Yin JC Xia G Xing M Chen X. Additive Effects of Variants of Unknown Significance in Replication Repair-Associated DNA Polymerase Genes on Mutational Burden and Prognosis Across Diverse Cancers. J Immunother Cancer (2021) 9. doi: 10.1136/jitc-2021-002336 PMC8420654 34479923

[30] Schrock AB Ouyang C Sandhu J Sokol E Jin D Ross JS. Tumor Mutational Burden Is Predictive of Response to Immune Checkpoint Inhibitors in MSI-High Metastatic Colorectal Cancer. Ann Oncol (2019) 30:1096–103. doi: 10.1093/annonc/mdz134 31038663

[31] Lizardo DY Kuang C Hao S Yu J Huang Y Zhang L. Immunotherapy Efficacy on Mismatch Repair-Deficient Colorectal Cancer: From Bench to Bedside. Biochim Biophys Acta Rev Cancer (2020) 1874:188447. doi: 10.1016/j.bbcan.2020.188447 33035640 PMC7886024

[32] Davis RJ Welcker M Clurman BE. Tumor Suppression by the Fbw7 Ubiquitin Ligase: Mechanisms and Opportunities. Cancer Cell (2014) 26:455–64. doi: 10.1016/j.ccell.2014.09.013 PMC4227608 25314076

[33] Davis H Lewis A Behrens A Tomlinson I. Investigation of the Atypical FBXW7 Mutation Spectrum in Human Tumours by Conditional Expression of a Heterozygous Propellor Tip Missense Allele in the Mouse Intestines. Gut (2014) 63:792–9. doi: 10.1136/gutjnl-2013-304719 PMC3995284 23676439

[34] Li Z Xiao J Hu K Wang G Li M Zhang J. FBXW7 Acts as an Independent Prognostic Marker and Inhibits Tumor Growth in Human Osteosarcoma. Int J Mol Sci (2015) 16:2294–306. doi: 10.3390/ijms16022294 PMC4346837 25622249

[35] Wang Z Inuzuka H Zhong J Wan L Fukushima H Sarkar FH. Tumor Suppressor Functions of FBW7 in Cancer Development and Progression. FEBS Lett (2012) 586:1409–18. doi: 10.1016/j.febslet.2012.03.017 PMC3372850 22673505

[36] Izumi N Helker C Ehling M Behrens A Herzog W Adams RH. Fbxw7 Controls Angiogenesis by Regulating Endothelial Notch Activity. PLoS One (2012) 7:e41116. doi: 10.1371/journal.pone.0041116 22848434 PMC3407154

[37] Tu K Yang W Li C Zheng X Lu Z Guo C. Fbxw7 Is an Independent Prognostic Marker and Induces Apoptosis and Growth Arrest by Regulating YAP Abundance in Hepatocellular Carcinoma. Mol Cancer (2014) 13:110. doi: 10.1186/1476-4598-13-110 24884509 PMC4035898

[38] Liu F Zou Y Wang F Yang B Zhang Z Luo Y. FBXW7 Mutations Promote Cell Proliferation, Migration, and Invasion in Cervical Cancer. Genet Test Mol Biomarkers (2019) 23:409–17. doi: 10.1089/gtmb.2018.0278 31161818

[39] Weng AP Ferrando AA Lee W Morris JP Silverman LB Sanchez-Irizarry C. Activating Mutations of NOTCH1 in Human T Cell Acute Lymphoblastic Leukemia. Science (2004) 306:269–71. doi: 10.1126/science.1102160 15472075

[40] Hodis E Watson IR Kryukov GV Arold ST Imielinski M Theurillat JP. A Landscape of Driver Mutations in Melanoma. Cell (2012) 150:251–63. doi: 10.1016/j.cell.2012.06.024 PMC3600117 22817889

[41] Akhoondi S Sun D von der Lehr N Apostolidou S Klotz K Maljukova A. FBXW7/hCDC4 Is a General Tumor Suppressor in Human Cancer. Cancer Res (2007) 67:9006–12. doi: 10.1158/0008-5472.CAN-07-1320 17909001

[42] Mao JH Perez-Losada J Wu D Delrosario R Tsunematsu R Nakayama KI. Fbxw7/Cdc4 Is a P53-Dependent, Haploinsufficient Tumour Suppressor Gene. Nature (2004) 432:775–9. doi: 10.1038/nature03155 15592418

[43] Boland CR Goel A. Microsatellite Instability in Colorectal Cancer. Gastroenterology (2010) 138:2073–2087.e2073. doi: 10.1053/j.gastro.2009.12.064 20420947 PMC3037515

[44] Lin A Zhang J Luo P. Crosstalk Between the MSI Status and Tumor Microenvironment in Colorectal Cancer. Front Immunol (2020) 11:2039. doi: 10.3389/fimmu.2020.02039 32903444 PMC7435056

[45] Zhang L Dong X Yan B Yu W Shan L. CircAGFG1 Drives Metastasis and Stemness in Colorectal Cancer by Modulating YY1/CTNNB1. Cell Death Dis (2020) 11:542. doi: 10.1038/s41419-020-2707-6 32681092 PMC7367849

[46] Bian J Dannappel M Wan C Firestein R. Transcriptional Regulation of Wnt/β-Catenin Pathway in Colorectal Cancer. Cells (2020) 9:2125–53. doi: 10.3390/cells9092125 PMC7564852 32961708

