# Supplementary figures and images for: FBXW7 and Its Downstream NOTCH Pathway Could be Potential Indicators of Organ-Free Metastasis in Colorectal Cancer
Source: Front Oncol. 2022 May 27;11:783564. doi: 10.3389/fonc.2021.783564 (PMC9197223; doi:10.3389/fonc.2021.783564)

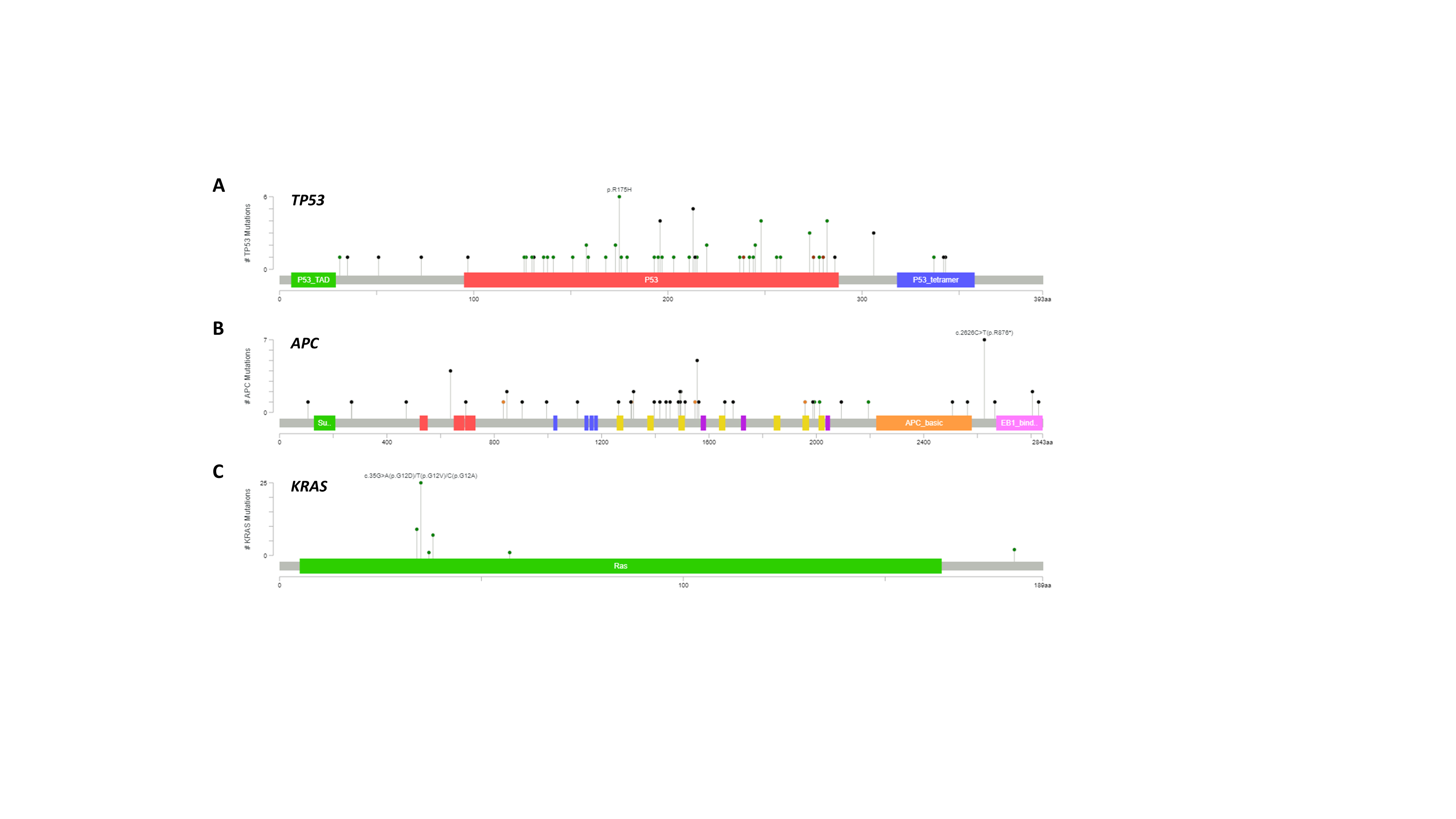

Supplement: Supplementary Figure 1 — The distribution of the detected somatic variants in this study. (A), TP53; (B), APC and (C), KRAS. [file Image_1.tif]

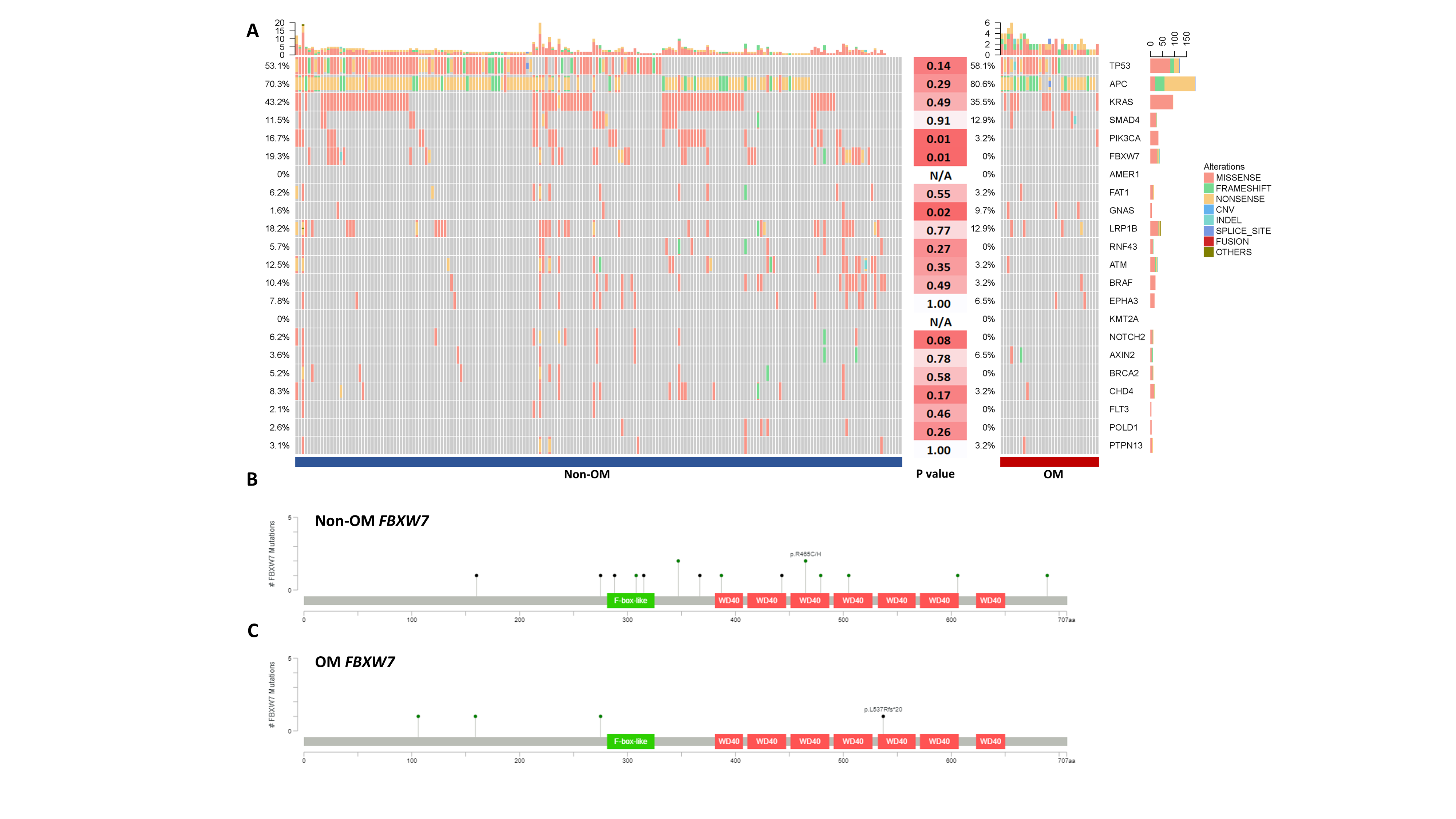

Supplement: Supplementary Figure 2 — (A) The mutational spectrum of the colorectal patients in TCGA cohort. Comparison of the mutational profile between the Non-OM and OM subgroup and the P values according to the Fisher’s exact test was shown. The distribution of the detected FBXW7 variants in the non-OM (B) and OM (C) groups. [file Image_2.tif]

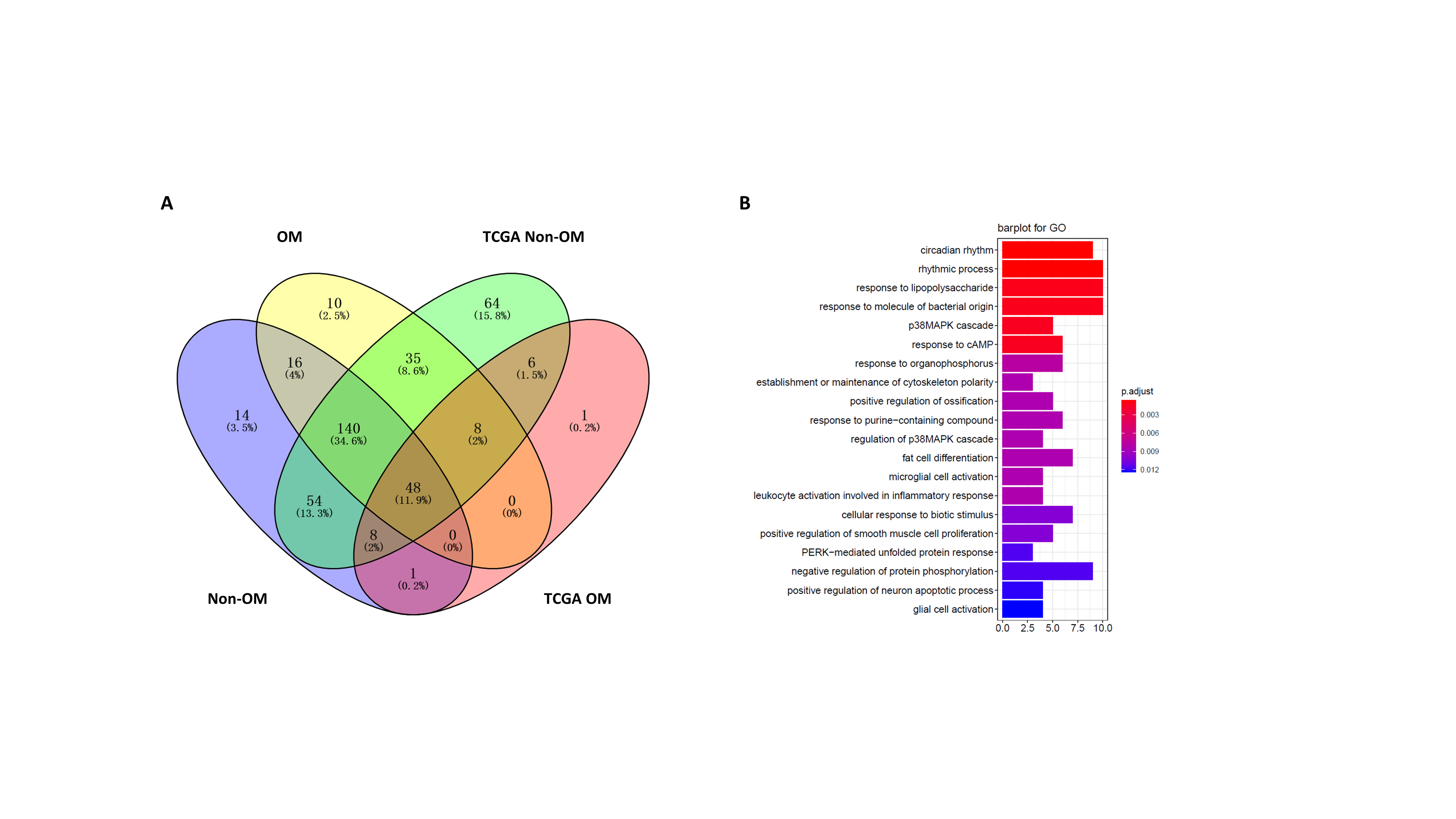

Supplement: Supplementary Figure 3 — The distribution of the mutated genes in both this study and TCGA cohort with or without organ metastasis (A). The GO pathway analysis of the 54 genes that only distributed in the OM groups (B). [file Image_3.tif]

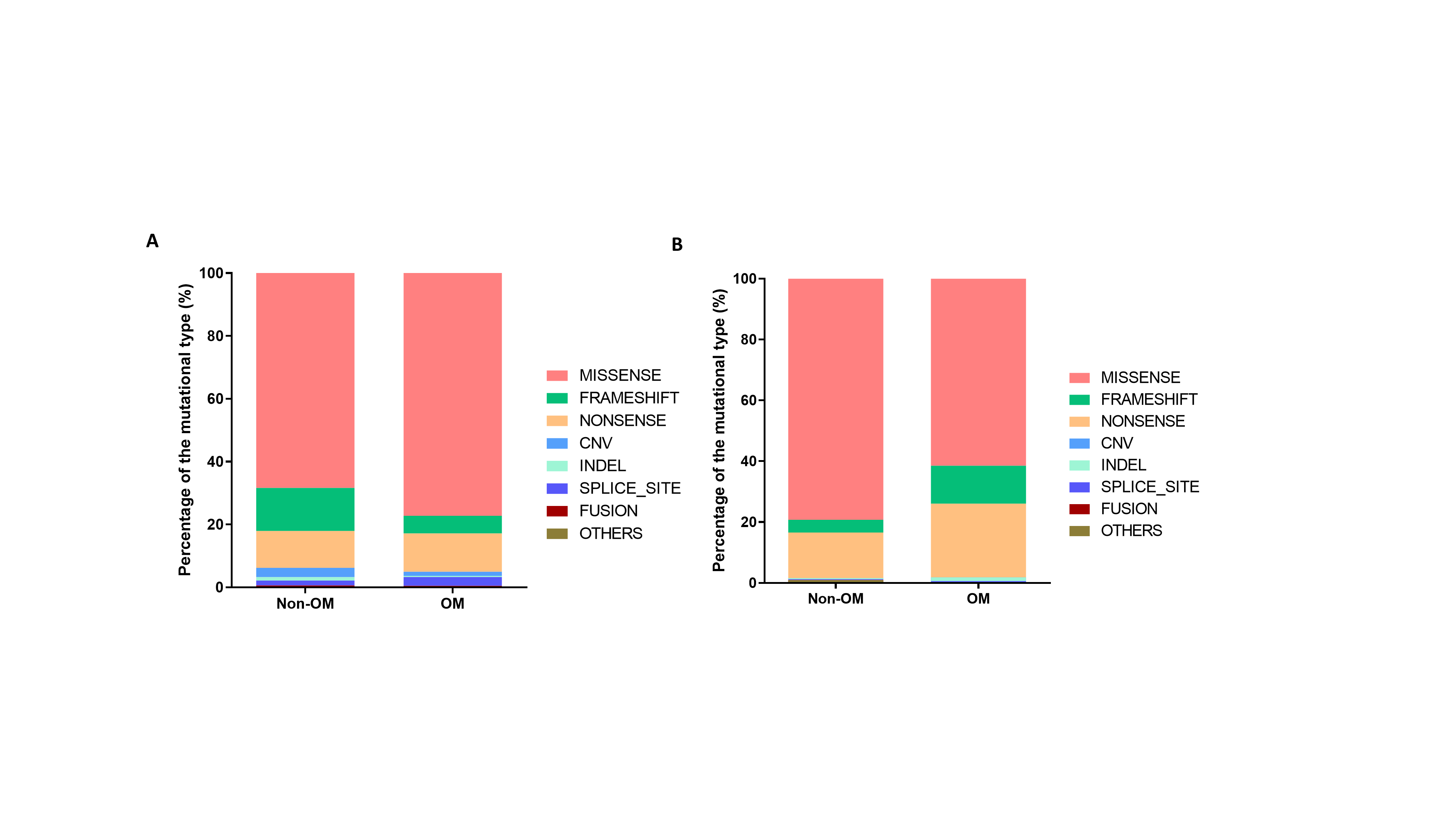

Supplement: Supplementary Figure 4 — The mutational type proportion of Non-OM and OM groups in this study (A) and TCGA cohort (B). [file Image_4.tif]

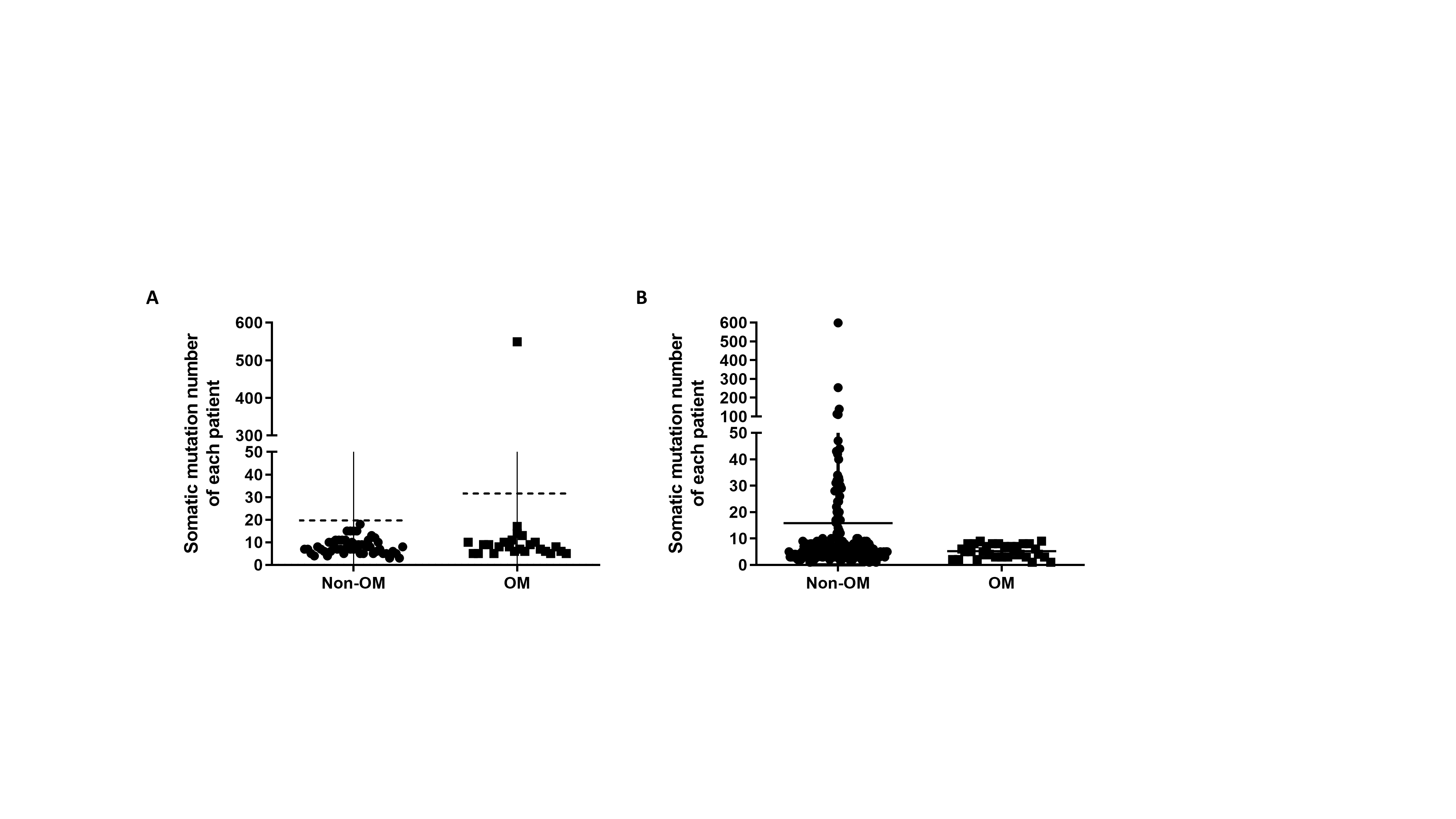

Supplement: Supplementary Figure 5 — The numbers of the somatic mutations of each patient in the Non-OM and OM groups of this study (A) and TCGA cohort (B). [file Image_5.tif]

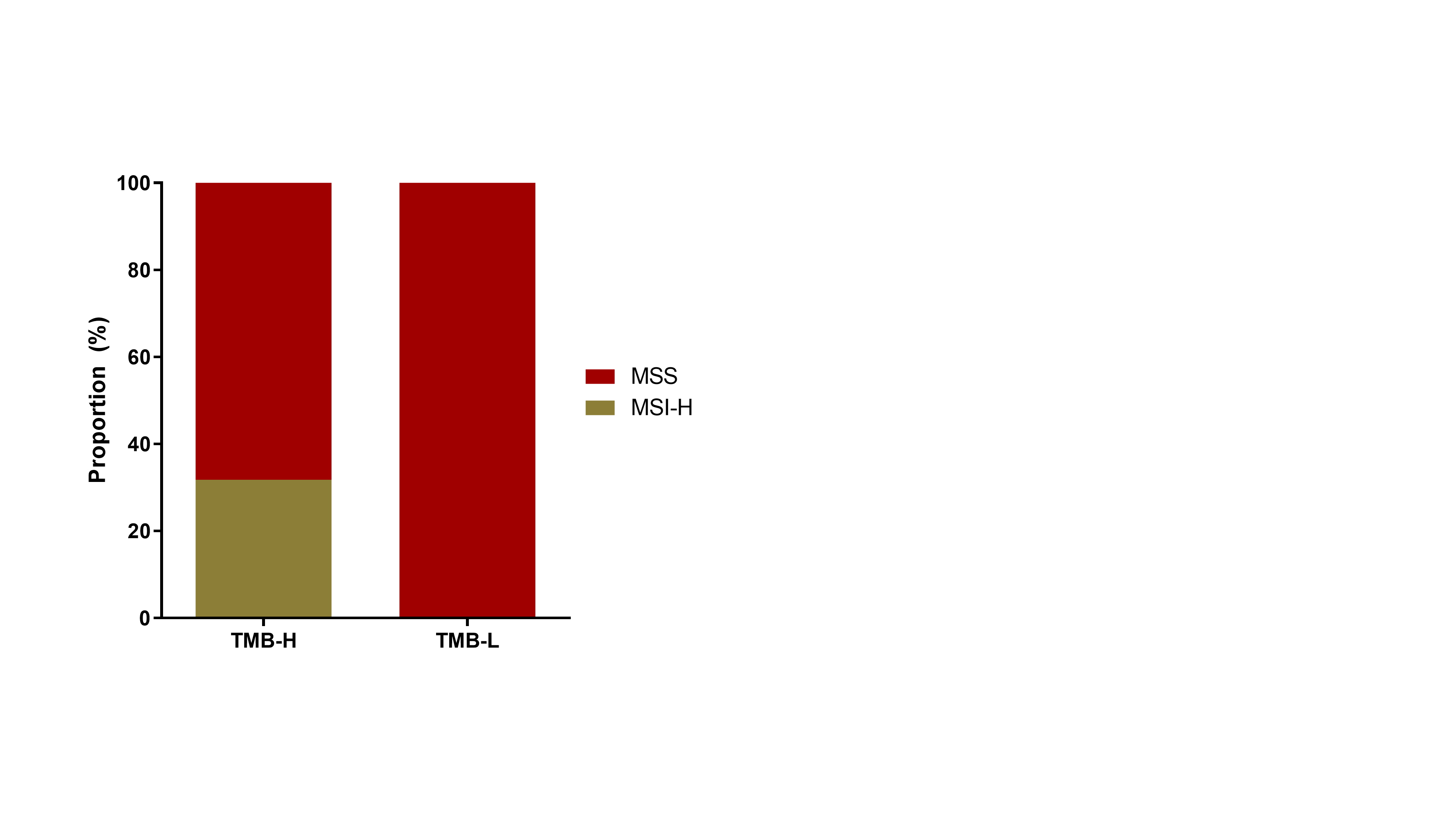

Supplement: Supplementary Figure 6 — The interaction of TMB and MS status in this study. [file Image_6.tif]

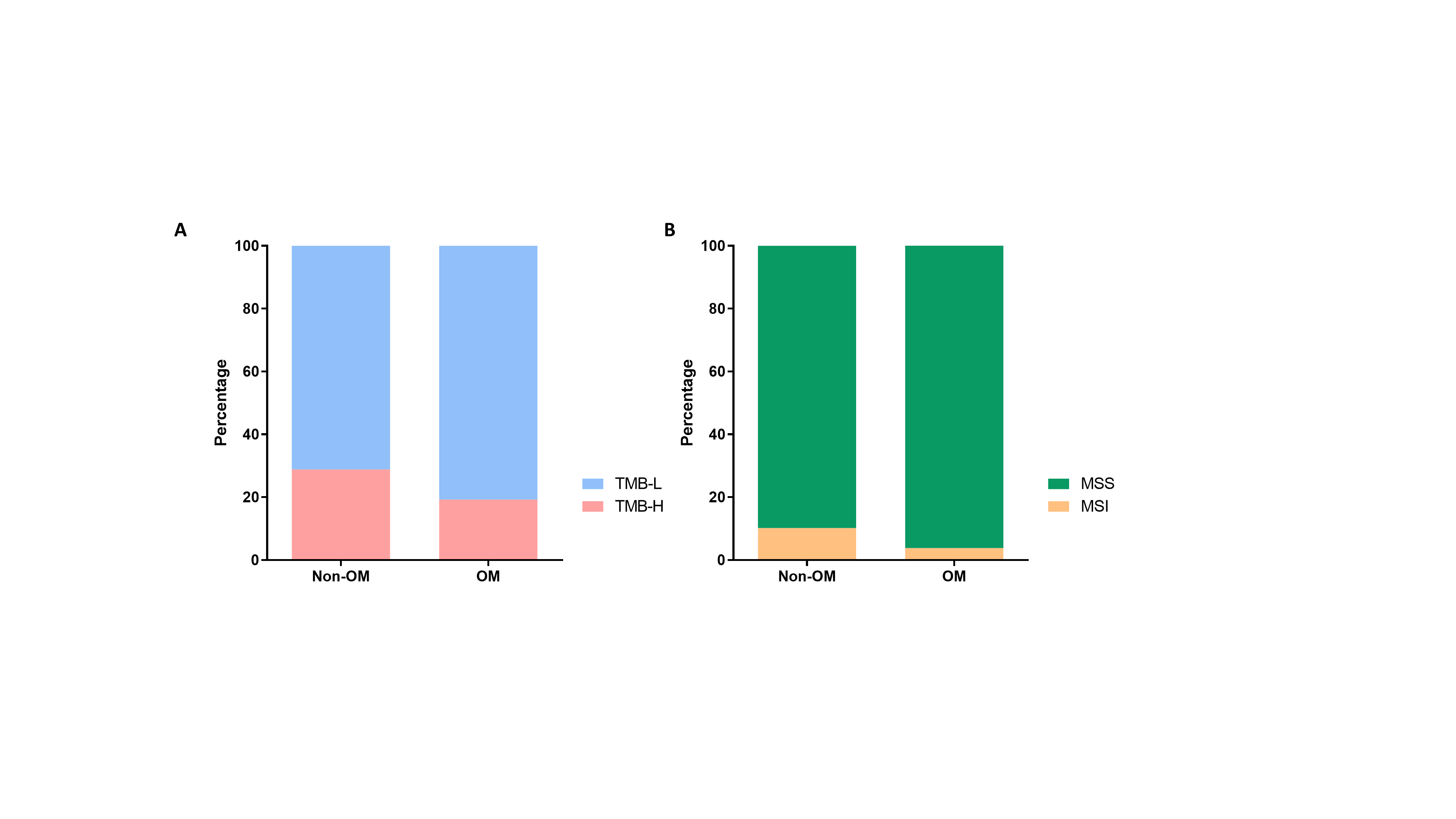

Supplement: Supplementary Figure 7 — The TMB (A) and MSI (B) distribution in the Non-OM and OM groups of this study. [file Image_7.tif]

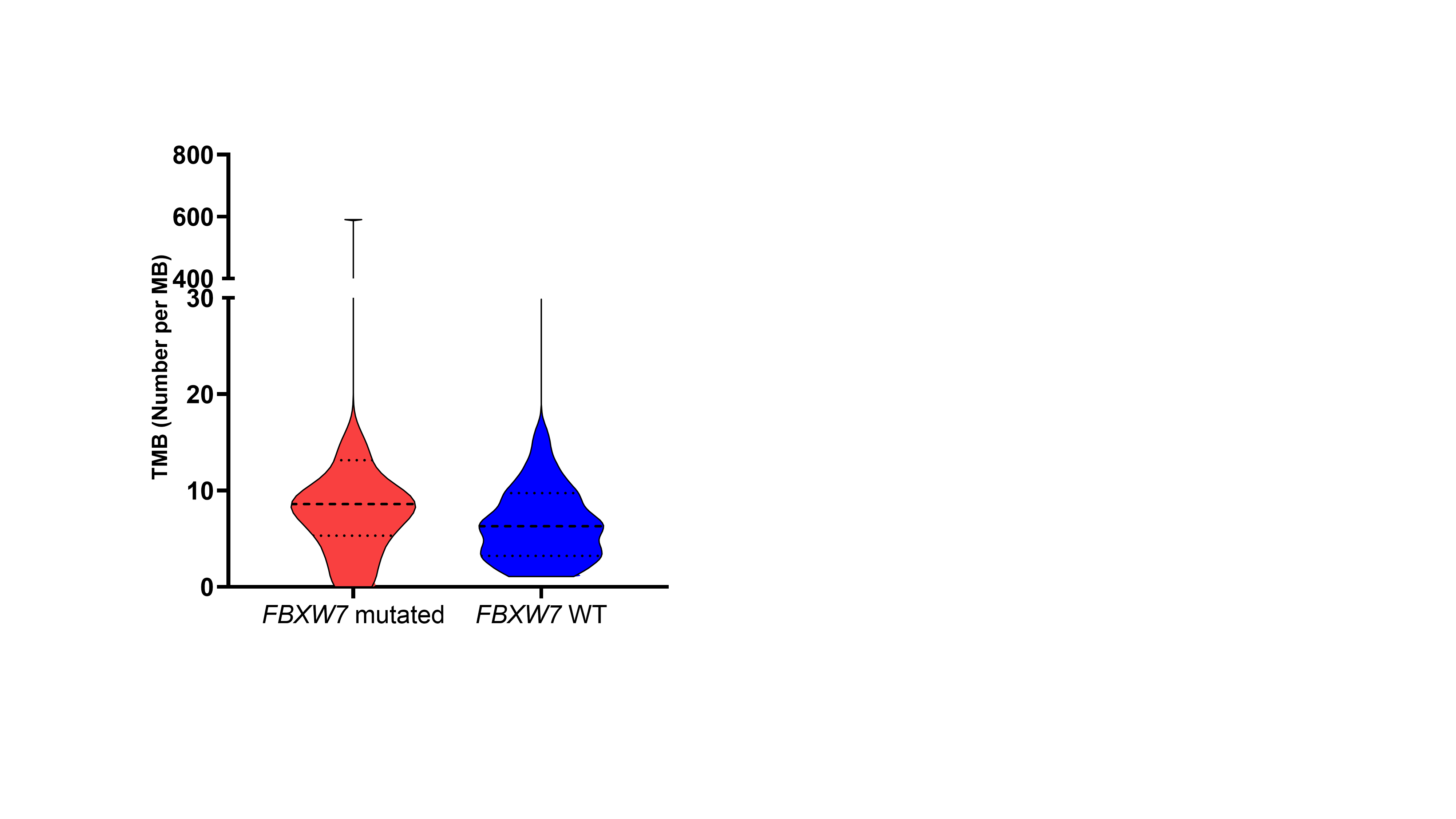

Supplement: Supplementary Figure 8 — The comparison of TMB between FBXW7 mutated and WT. [file Image_8.tif]
