# Supplementary material for: FBXW7 and Its Downstream NOTCH Pathway Could be Potential Indicators of Organ-Free Metastasis in Colorectal Cancer
Source: Front Oncol. 2022 May 27;11:783564. doi: 10.3389/fonc.2021.783564 (PMC9197223; doi:10.3389/fonc.2021.783564)
Supplement: Supplementary file 9 [file DataSheet_1.docx]

**Supplementary Table 1. Significantly altered genes between non-lymph metastasis and lymph metastasis.**

| **Gene name** | **Non-lymph metastasis** | **Lymph metastasis** | **P value** |
| --- | --- | --- | --- |
| CHEK1 | 21.43% | 0.00% | <0.01 |
| NTRK3 | 25.00% | 1.79% | <0.01 |
| SETD2 | 25.00% | 1.79% | <0.01 |
| ATM | 28.57% | 3.57% | <0.01 |
| CYSLTR2 | 17.86% | 0.00% | <0.01 |
| PDCD1 | 17.86% | 0.00% | <0.01 |
| SETBP1 | 17.86% | 0.00% | <0.01 |
| ALK | 21.43% | 1.79% | <0.01 |
| DDR2 | 21.43% | 1.79% | <0.01 |
| IGF1R | 21.43% | 1.79% | <0.01 |
| PKHD1 | 21.43% | 1.79% | <0.01 |
| POLE | 21.43% | 1.79% | <0.01 |
| QKI | 21.43% | 1.79% | <0.01 |
| CHD4 | 25.00% | 3.57% | 0.01 |
| CBL | 14.29% | 0.00% | 0.01 |
| EXT2 | 14.29% | 0.00% | 0.01 |
| FLT4 | 14.29% | 0.00% | 0.01 |
| JARID2 | 14.29% | 0.00% | 0.01 |
| KDM5A | 14.29% | 0.00% | 0.01 |
| MSH2 | 14.29% | 0.00% | 0.01 |
| PALB2 | 14.29% | 0.00% | 0.01 |
| POLH | 14.29% | 0.00% | 0.01 |
| PTCH1 | 14.29% | 0.00% | 0.01 |
| PTK2 | 14.29% | 0.00% | 0.01 |
| WRN | 14.29% | 0.00% | 0.01 |
| AMER1 | 32.14% | 8.93% | 0.01 |
| TP53 | 67.86% | 91.07% | 0.01 |
| KMT2A | 25.00% | 5.36% | 0.01 |
| NOTCH2 | 25.00% | 5.36% | 0.01 |
| AR | 17.86% | 1.79% | 0.01 |
| BLM | 17.86% | 1.79% | 0.01 |
| CREBBP | 17.86% | 1.79% | 0.01 |
| CTCF | 17.86% | 1.79% | 0.01 |
| DOT1L | 17.86% | 1.79% | 0.01 |
| EPHA5 | 17.86% | 1.79% | 0.01 |
| FANCA | 17.86% | 1.79% | 0.01 |
| NRG1 | 17.86% | 1.79% | 0.01 |
| PIK3R1 | 17.86% | 1.79% | 0.01 |
| RAD50 | 17.86% | 1.79% | 0.01 |
| RPTOR | 17.86% | 1.79% | 0.01 |
| ATR | 21.43% | 3.57% | 0.01 |
| KMT2B | 21.43% | 3.57% | 0.01 |
| PTEN | 21.43% | 3.57% | 0.01 |
| AKT3 | 10.71% | 0.00% | 0.03 |
| B2M | 10.71% | 0.00% | 0.03 |
| CDC73 | 10.71% | 0.00% | 0.03 |
| CYLD | 10.71% | 0.00% | 0.03 |
| EPHA2 | 10.71% | 0.00% | 0.03 |
| ESR1 | 10.71% | 0.00% | 0.03 |
| FANCC | 10.71% | 0.00% | 0.03 |
| IFNGR1 | 10.71% | 0.00% | 0.03 |
| JAK2 | 10.71% | 0.00% | 0.03 |
| LZTR1 | 10.71% | 0.00% | 0.03 |
| MAP2K2 | 10.71% | 0.00% | 0.03 |
| MAP2K4 | 10.71% | 0.00% | 0.03 |
| MEF2B | 10.71% | 0.00% | 0.03 |
| MLH1 | 10.71% | 0.00% | 0.03 |
| MRE11 | 10.71% | 0.00% | 0.03 |
| MSH6 | 10.71% | 0.00% | 0.03 |
| NTRK1 | 10.71% | 0.00% | 0.03 |
| NTRK2 | 10.71% | 0.00% | 0.03 |
| PLCB4 | 10.71% | 0.00% | 0.03 |
| PMS1 | 10.71% | 0.00% | 0.03 |
| PRKCI | 10.71% | 0.00% | 0.03 |
| PTPN11 | 10.71% | 0.00% | 0.03 |
| TOP2A | 10.71% | 0.00% | 0.03 |
| CHD8 | 17.86% | 3.57% | 0.04 |
| ERCC5 | 17.86% | 3.57% | 0.04 |
| GRIN2A | 17.86% | 3.57% | 0.04 |
| NBN | 17.86% | 3.57% | 0.04 |
| PBRM1 | 17.86% | 3.57% | 0.04 |
| SMARCA4 | 17.86% | 3.57% | 0.04 |
| ARAF | 14.29% | 1.79% | 0.04 |
| FANCM | 14.29% | 1.79% | 0.04 |
| FLCN | 14.29% | 1.79% | 0.04 |
| NOTCH1 | 14.29% | 1.79% | 0.04 |
| RAF1 | 14.29% | 1.79% | 0.04 |
| ROS1 | 14.29% | 1.79% | 0.04 |
| SDHA | 14.29% | 1.79% | 0.04 |

**Supplementary Table 2. All *FBXW7* variants detected in this study**

| **DNA change** | **AA change** | **Mutation classification** | **OncoKB annotation** | **Group** |
| --- | --- | --- | --- | --- |
| c.1394G>A | p.R465H | Missense | Oncogenic/likely oncogenic | non-OM |
| c.2065C>T | p.R689W | Missense | Oncogenic/likely oncogenic | non-OM |
| c.1099C>T | p.R367* | Nonsense | Oncogenic/likely oncogenic | non-OM |
| c.1159C>T | p.R387C | Missense | Likely benign/Unknown | non-OM |
| c.985+1G>T | - | Splice site | Oncogenic/likely oncogenic | non-OM |
| c.2122_*2delTGAAG | - | UTR | Likely benign/Unknown | non-OM |
| c.943del | p.I315Sfs*65 | Frameshift | Oncogenic/likely oncogenic | non-OM |
| c.1513C>T | p.R505C | Missense | Oncogenic/likely oncogenic | non-OM |
| c.1817G>T | p.W606L | Missense | Likely benign/Unknown | non-OM |
| c.1436G>A | p.R479Q | Missense | Oncogenic/likely oncogenic | non-OM |
| c.1393C>T | p.R465C | Missense | Oncogenic/likely oncogenic | non-OM |
| c.1039C>T | p.R347C | Missense | Likely benign/Unknown | non-OM |
| c.478C>T | p.R160* | Nonsense | Oncogenic/likely oncogenic | non-OM |
| c.823C>T | p.R275* | Nonsense | Oncogenic/likely oncogenic | non-OM |
| c.923C>T | p.S308L | Missense | Likely benign/Unknown | non-OM |
| c.864G>A | p.W288* | Nonsense | Oncogenic/likely oncogenic | non-OM |
| c.1328del | p.T443Nfs*17 | Frameshift | Oncogenic/likely oncogenic | non-OM |
| c.317G>A | p.R106Q | Missense | Likely benign/Unknown | OM |
| c.475C>A | p.Q159K | Missense | Likely benign/Unknown | OM |
| c.824G>A | p.R275Q | Missense | Likely benign/Unknown | OM |
| c.1606_1607dupAC | p.L537Rfs*20 | Frameshift | Oncogenic/likely oncogenic | OM |
